# European psychotraumatology – alongside the recent European history

**DOI:** 10.3402/ejpt.v4i0.21304

**Published:** 2013-06-06

**Authors:** Brigitte Lueger-Schuster

**Affiliations:** Faculty of Psychology, University of Vienna, Vienna, Austria

**Keywords:** Trauma, PTSD, European psychotraumatology, ESTSS

## Abstract

This article outlines a personal reflection of experiences within the field of traumatic stress, especially in relation to specific events, which affected the author's professional life. Conclusions for further challenges for European Society for Traumatic Stress Studies (ESTSS) are delineated. ESTSS's role in the global network of traumatic stress societies is discussed. This is a personal view of Brigitte Lueger-Schuster, president of ESTSS on behalf of the 20th birthday of ESTSS.

Growing up in the Austrian Alps is not a predictable background for a European presidency, leading to a window of opportunity for chairing the European Society dealing with traumatic stress. Growing up in the Alps predicts becoming good at skiing. Despite this, between 2011 and 2013 I have been the president of European Society for Traumatic Stress Studies (ESTSS), a challenging and very inspiring position. In my contribution, I will refer to some experiences of my own professional development and use them to identify European issues and future challenges for European Psychotraumatology.

## First steps in psychotraumatology – refugees from the Balkans

In the early 1990s, the former Republic of Yugoslavia turned into a battleground. The war in Yugoslavia turned 100,000s of individuals into refugees, some stranded in Vienna. At that time, the University of Vienna offered shelter to up to 300 refugees in a former University hospital. Together with a native-speaking psychologist, we learned about the needs of the refugees; we tried to provide structures to enhance their feeling of security in order to stabilize their emotions. We developed many services, such as a kindergarten, talking groups for women, learning assistance for pupils, individual treatment, integrated more and more students and became highly involved and stressed, indeed very stressed. We tried to find a supervisor; we tried to learn from former experiences of refugee crisis around Vienna (1945—survivors of the holocaust and displaced persons; 1956—Hungaria; 1968—Czechoslovakia; 1980s—Poland), but most of the knowledge was gone or only for individual treatment and thus not appropriate for a large-scale experience.

Surprisingly, a country like Austria with a traumatic history from two World Wars, in which it was involved both as perpetrator and victim, could not provide a tradition of dealing with survivors of war and refugees, but the same was true for the European continent. Besides the practical work, I wrote a book (Lueger-Schuster, 1996), addressing specific issues such as women, children and torture, which we encountered. Later, having established contacts with psychiatrists providing services for holocaust survivors, an Austrian working group in psychotraumatology was formed, aiming to connect the pioneers in the field, exchange experiences, and provide information on PTSD, its assessment and treatment of students and colleagues. These issues were integrated into another book (Friedmann et al., 2004). My research topic was still connected with refuge issues and looking around Europe for researchers working in the same area.

One of the first international people in psychotraumatology I met was Andreas Maercker. This encounter brought me to a conference of the German Speaking Society of Traumatic Stress (DeGPT) in Jena. There I was invited for a business meeting and asked to join the board as Austrian representative. From 2000 to 2006, I was a member of the board and supported the development of German-speaking activities. One of our core goals was the development and implementation of evidence-based certified psychotrauma–psychotherapy. Very successfully, a network of training institutes was certified by the society, which brought a large number of members to it.

## Acute psychosocial support

Parallel to the German-speaking activities, I became involved in the civil protection unit of the City Government of Vienna who wanted to establish an acute psychosocial support unit for disaster situations as one of the consequences from the lessons learned out of the refugee crisis around the former Yugoslavian war. In 1995, I started to work on a concept, addressing the needs of individuals with exposure to traumatic stress situations in the acute phase of coping. The idea was to provide for survivors a “communication space” to learn more about the exposure and their immediate reactions to it, combined with practical help, e.g., administrative processes after the sudden death of a family member or housing problems, when homes are damaged. A network of specific mental health care centers gave a further transfer into treatment after a phase of watchful waiting. International contacts were established and this is how I got to know Berthold Gersons.

By 1998, we had created a group of well-trained psychologists, psychotherapists, nurses and social workers who offered support for refugees coming from Kosovo. In this period, other support units for trauma were created, for example, by the Red Cross. An Austrian platform for acute psychosocial support after traumatic exposure was established, the Austrian activities were presented in several European disaster management conferences and finally Vienna decided to organize a European civil protection conference for acute psychosocial support. Inspired by this, Belgium took the initiative to develop further guidelines for acute psychosocial support (Seynaeve et al., 2004).

From my practical experience of acute support, I have learnt that parents and children have a common problem in coping. The children wanted to know what happened and why everybody was stressed and unhappy, and the parents wanted to protect the children from further harm. Together with Christine Nöstlinger, who designed the drawings for her mother's children's books (winning the Astrid Lindgren award), we wrote the books of Pippa (for affected children, parents and helpers). Pippa is a girl who has to cope with trauma, an evidence-informed story with examples of how to cope and translated into a children's world with pictures including Leorix, the lion lending her support (Lueger-Schuster, & Pal-Handl, 2004; Pal-Handl, Lackner, & Lueger-Schuster, 2004).

In 2004, a tsunami hit Asian coasts and Austria was affected by the hundreds of Austrians both missing and dead. Vienna gave psychosocial support to the families and friends affected following European Guidelines (Seynaeve et al., 2004). I guided the team through the longest acute support phase ever, ending when the last Viennese body was identified in Asia in August 2005. Typically acute grief turned into complex grief and traumatic memory interfered with coping and triggered further symptoms associated with PTSD and somatic complaints. The focus of support was the stabilization of symptoms by psychoeducation, emotion ventilation, cognitive restructuring and practical support. A brief report was published in the European Bulletin in 2005 (Lueger-Schuster, 2005) and we subsequently wrote another book for disaster situations, specifically addressing the needs of affected persons within the acute phase (Lueger-Schuster, Kürsmann, & Purtscher, 2006).

## Man-made disaster—the Siege in Beslan

In 2004, the Siege in Beslan took place and I was appointed to deliver the Austrian support together with other Austrian experts. We decided to provide a center for young survivors and also indirectly affected young people. We provided training in the region for nurses, physicians, psychologists and social workers, we went through the bombed school, we met the mothers, and finally a group of experts from Beslan came to Vienna. We followed the goal of reaching stabilization via social support and the modification of structures and trauma-informed personnel who provide comprehensive services for the injured survivors. Most impressive was the degree of connectedness of families within the region of Beslan. It had not only been the families who lost beloved ones, who suffered from grief and traumatic stress. Grief and stress spread over the whole area and triggered other traumatic memories. Besides training for working in a centre for the youth of Beslan, we provided supervision, covering casework, the triggers, and the rivalry between directly and indirectly affected children experiences (Lueger-Schuster, 2011). Acknowledgment from other experts was considered to be the most important factor experienced by the local experts. Coming from far away, making a really difficult journey several times over an extended period was seen and understood as a major indicator of support for them.

## ESTSS career

In 2007, I ran as candidate for the ESTSS board, as I was encouraged to do so by Dean Ajdukovic, Berthold Gersons and Jon Bisson. I joined the board and took over responsibility for the website as its editor. I was part of the two TENTS projects (Ajdukovic, 2013; Bisson, 2013; Olff, 2013; Pearce et al., 2012; Witteveen et al., 2012). Miranda Olff followed Jon Bisson as president; she established the STSS network of national societies and developed the EJPT. These two major projects were being supported by the small shoulders of the ESTSS administration when I was asked to serve as president, given my DeGPT background and leadership qualities. What a flattering offer and what a challenge! My objectives have been the consolidation of the administration and to establish a young minds initiative. I became president elect in 2010 and had 1 year to organize ECOTS 2011, the last ECOTS (ECOTS was renamed “ESTSS Conference” to stress that ECOTS has always been the ESTSS conference).

Following my belief that traumatic stress and coping with traumatic stress needs much more than treatment, I focused ECOTS 2011 on psychotraumatology and human rights (Ørner, 2013). Almost 1,000 participants from all over the world enjoyed Vienna, the conference and Wiener Schnitzel. It was the first time that ESTSS had organized a conference jointly with a national society (the German-speaking society) and a parallel conference with EMDR. The joint organization will be repeated in Bologna and in further conferences. It offers the opportunity to have some symposia in the mother tongue, in addition to learning from the international scientists who always gather at the ESTSS conferences.

## Future challenges for ESTSS

When ESTSS was turned into an umbrella organization, the structure turned into a communication challenge. Members of the national societies have to register to join ESTSS; this is communicated by the national societies which follow their own protocols when communicating with their members. The national presidents are informed about any news, as are all the members. The national presidents are invited to frequently communicate their ideas, needs and feedback to the ESTSS president. All of us are volunteers, having primary jobs and ESTSS adds another slice of work and duty. Additionally some of us are double agents, representing national interests on the one hand and being ESTSS board members on the other hand. This can bring intrapersonal conflicts into daily business, national interests might win the race against ESTSS interests, but all of this is minor compared to the joy of sharing interests with a group of colleagues.

There is, however, a need for stronger administrative support in order to meet these communication challenges. Most of the national societies have the feeling that they profit from being part of ESTSS but there are challenges. Sometimes a language barrier prevents effective communication. The board structure and the role of the national presidents need further attention. It is important that they are all optimally involved in the decisions being made. Ultimately, however, ESTSS is valued as having facilitated the development of psychotraumatology and it is nice to be a part of this.

So what is the core business of ESTSS beside the mission described on the website? First, it is the delivery of high-class conferences and workshops; second, it helps national issues to have a European organization in the background; and third, it is a network for people sharing goals, providing peer support and friendship by collaborating in research and practical issues. Putting together manualized evidenced-based treatments and further tools for intervention might be a future challenge for European Psychotraumatology, thus getting closer to affected individuals. A way into individual treatment might be opened for those who are at first unwilling to see a therapist. Having a therapist is unusual and not as common as it is in the United States. Providing tools to help those individuals who refuse therapy, especially for the eastern part of Europe, where mental health problems are still more stigmatized should be adressed in the future by ESTSS.

The classification of PTSD is also crucial for the future of European Psychotraumatology. Having undertaken assessments with refugees, torture survivors and survivors of different kinds of disaster (both natural and man-made), the current classifications have turned out to be insufficient for all their needs, reactions and symptoms. To cope better with the assessment, a project such as “Children of the War in Austria”, reflecting the childhood exposure of World War II in Austria (Gluck, Tran, & Lueger-Schuster, 2012; Lueger-Schuster, Glück, Tran, & Zeilinger, 2012; Tran, Gluck, & Lueger-Schuster, 2013) integrating all European countries might help to compile missing symptoms for groups with a history of traumatic exposure, often over a long period of time. To consider the political development in European countries in research and practice, the long-term effects of living conditions connected with, e.g., an iron curtain, separating the continent for more than 40 years or several regimes, established or lasting after World War II in the south of Europe, ESTSS might stimulate activities (workshops, conferences) in its national societies as future challenges. The issue of reconciliation is a topic to be addressed as trauma might interfere with the capacity to reconcile with the past (Ajdukovic, 2013).

A European-wide epidemiology of PTSD, considering the different political conditions over the European continent, or culturally sensitive clinical studies going beyond classical treatment studies by integrating new ideas to attract therapy-shy clients do not exist, and are missed. The results from such work might be transformed into training tools, respecting the specific national regulation for clinical work, but always adding evidence-based psychotraumatological competence.

The profile of ESTSS has always been less streamlined compared to the profile of ISTSS, which cannot deny its military background, rooted in the development of PTSD that was related to veterans from Vietnam. It is ESTSS's added value to be multi-faceted and colourful, truly international and inclusive in terms of its members and attendance at its conferences and members. ISTSS has international reputation on its board and has started with a global initiative to become more international. However, it still remains very American in its outlook (Gersons, 2013; Schnyder, 2013). As president of ESTSS, I was part of the ISTSS board and admired the professional attitude to their scientific work (Olff, 2013; Turner, 2013). However, I also picked up some members’ resistance to the internationalization of the organization, such as giving voting rights to the presidents of the ISTSS affiliated societies when they work for them on their board. I had two informative years as an ex-officio board member of ISTSS, challenged by the situation that ESTSS presidents are integrated in the board but have nothing but their voice to influence ISTSS decisions in the global issues, affecting both societies.

We are on our way to coping with the problem of having two large traumatic stress societies (ESTSS and ISTSS, Gersons, 2013) with long traditions of struggling with a common route (Ørner, 2013; Stuart, 2013) and have found a benign way of co-existence on the territory of Europe, namely by offering joint activities with joint promotion of the two societies. Furthermore, ESTSS is nicely integrated on the global initiative that was started by ISTSS (Olff, 2013; Schnyder, 2013). Nevertheless, the relationship between ESTSS and ISTSS has not been formalized in a structure which might indicate that ISTSS is the mother society of the other societies representing continents of the world. ESTSS and ISTSS work together respectfully and fruitfully since they represent different approaches to the consequences of traumatic stress. To develop the co-existence further and to strengthen the cooperation of both societies, in a way that is beneficial for all their members, will be challenging for the president elect, Vedat Sar. The global project, a direct output of the global initiative offers a wonderful opportunity for these future challenges (Schnyder & Olff, 2013).

When this contribution is published, I shall be in my final days as president with 1 year left until I am the past-president. This year will help us to consolidate the administration of ESTSS and help the new president with the communication challenge as mentioned above.

ESTSS being 20 years old is wonderful; being part of ESTSS in different ways has always given me an inspiring resource to cope with daily business in the academic world. ESTSS has provided me a professional home for many years, and hopefully for many others as well. I am a member of the second generation, not having been part of the group of founders and pioneers, but have always felt welcomed by them. The founders formed an integrative and inclusive basis for their successors and confidently, on this basis, I predict that the ESTSS will last much longer than the next 20 years. Happy Birthday!!
